# Electrochemical determination of tetrabromobisphenol A in water samples based on a carbon nanotubes@zeolitic imidazole framework-67 modified electrode†

**DOI:** 10.1039/c9ra06980a

**Published:** 2020-01-10

**Authors:** Tingting Zhou, Xiaoya Zhao, Yinghua Xu, Yun Tao, Dan Luo, Liqin Hu, Tao Jing, Yikai Zhou, Peng Wang, Surong Mei

**Affiliations:** Department of Clinical Laboratory, The Affiliated Hospital of Qingdao University #16, Jiangsu Road Qingdao Shandong 266003 China; Technology Center of Wuhan Customs Wuhan Hubei 430050 China 18681533@qq.com; State Key Laboratory of Environment Health (Incubation), Key Laboratory of Environment and Health, Ministry of Education, Key Laboratory of Environment and Health (Wuhan), Ministry of Environmental Protection, School of Public Health, Tongji Medical College, Huazhong University of Science and Technology #13 Hangkong Road Wuhan Hubei 430030 China

## Abstract

Carbon nanotubes@zeolitic imidazole framework-67 (CNTs@ZIF-67), a conductive composite was prepared from carboxylic carbon nanotubes and a cobalt–imidazole framework. It possesses an excellent adsorption capacity (92.12 mg g^−1^) for the flame retardant tetrabromobisphenol A (TBBPA). The composite was characterized by transmission and scanning electron microscopy, FTIR and X-ray diffractometry. It was then used to modify an acetylene black electrode. Electrochemical studies showed the current response of the modified electrode to be larger than that of electrodes modified with CNTs-COOH or ZIF-67 only. Electrochemical impedance spectroscopy showed this material combination to improve the conductivity of ZIF-67. The addition of perfluorodecanoic acid further improves the response. The sensor is stable, reproducible, and has a linear range of 0.01–1.5 μM TBBPA concentration, with a 4.2 nM detection limit (at S/N = 3). The sensor was successfully applied to the detection of TBBPA in spiked rain and pool water samples.

## Abstract 2

Carbon nanotubes@zeolitic imidazole framework-67 (CNTs@ZIF-67), a conductive composite was prepared from carboxylic carbon nanotubes and a cobalt–imidazole framework.

## Graphical abstract

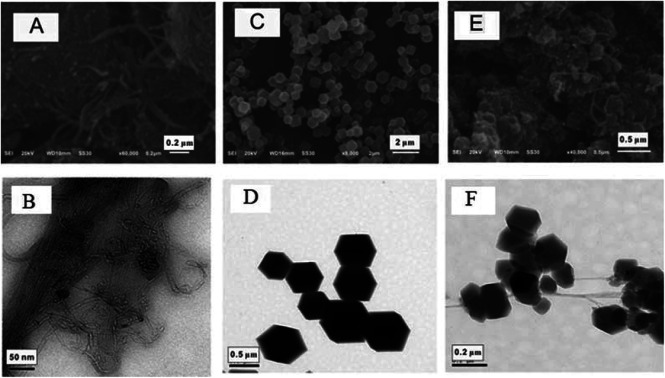

## 1. Introduction

Tetrabromobisphenol A (TBBPA) accounts for approximately 60% of commercially available brominated flame retardants, and its utilization is not restricted in many countries, such as Japan, the USA and China.^2,3^ However, reports illustrated that TBBPA is a relatively typical persistent organic pollutant,^1^ and it has been detected in numerous environmental samples, including dust, surface water, soil, sediment, air, sewage,^4–6^ and even in human urine, plasma, and breast milk.^7,8^ Even at environmentally relevant concentrations, TBBPA can cause a set of adverse effects for human health, such as endocrine disruption, immunity dysfunction, and neurotoxicity,^9,10^ especially thyroid hormone disruption.^11^ TBBPA usually gains access to the human body *via* water, so the determination of TBBPA in environmental water samples is essential for human health. Hence, rapid and sensitive detection of TBBPA in environmental water samples is of great important.

To date, different kinds of analytical methods have been used in the detection of TBBPA. Electrochemical detection methods are more advantageous than chromatography^12^ and chromatography-mass spectrometry^13^ in terms of speed, handling convenience, and cost. Several carbon materials, such as acetylene black (AB),^14,15^ carbon nanotubes (CNTs),^16^ and nitrogen-doped graphene,^17^ have been employed to construct electrochemical sensors for the detection of TBBPA. Although the carbon materials exhibit excellent conductivity, their adsorption capacity for TBBPA is insufficient, which decreases their sensitivity for detecting TBBPA. Metal organic frameworks (MOFs) have drawn much attention in electrochemical sensing due to their high specific surface area, adjustable pore size, and low density.^18–21^ Recently, several groups are trying to prepare MOF-derived carbons to enhance the stability.^22–25^ However, most MOFs present the drawback of low conductivity.^26^ To solve this problem, the use of semi-conductive material coupled to conductive one was appeared. What is more, the prepared composite was used in the determination of phenol like analytes.^27–32^ In the field of electrochemical, MOF-based electrodes were usually modified with conductive materials, such as Au nanoparticles^33^ and Nafion® ion exchangers.^34^ Though the performance of carbon materials in improving the electrode conductivity is preferable, the application of carbon materials to the preparation of MOF-based sensors is still limited. To the best of our knowledge, only the composite of Ni(ii)-MOF@CNTs has been prepared by Wang *et al.*^35^ and was finally used in the electrochemical determination of H_2_O_2_.

Zeolitic imidazole framework-67 (ZIF-67), a typical kind of MOF, possesses promising adsorption capability and high surface area, has been used in the removal of organic pollutants in aqueous environments.^36^ However, the utilization of ZIF-67 in the development of electrochemical sensors has yet to be investigated. In this work, a novel CNTs@ZIF-67 composite was prepared with the addition of CNTs. It was then used as an adsorption and recognition element in the construction of a sensitive detector for TBBPA.

## 2. Experimental section

### 2.1 Chemicals and materials

TBBPA (99%), tetrabromobisphenol A-bis(dibromopropyl ether) (TBBME, 98%), tetrachlorobisphenol A (TCBPA, 98%), hexafluorobisphenol A (BPAF, 98%), bisphenol A (BPA, 99%), 4,4-sulphonyl-bis-(2,6-dibromophenol) (TBBPS, 98%) and tetrabromobisphenol A diallyl ether (TBBDE, 99%) were obtained from Shanghai Meryer Chemical Technology Company. The chemical structures of TBBPA and its analogs are displayed in Fig. S1 (see ESI†). Perfluorobutanoic acid (PFUA, 98%), perfluorooctanoic acid (PFOA, 98%), perfluoroundecanoic acid (PFUnDA, 96%), perfluorodecanoic acid (PFDA, 98%), perfluorononanoic acid (PFNA, 97%), perfluorododecanoic acid (PFDoDA, 95%), perfluorotetradecanoic acid (PFTA, 97%), perfluorodecanesulfonic acid (PFD-SO_3_, 98%), and perfluoro-1-decanol (PFD-OH, 99%) were purchased from J&K Scientific Company. (Beijing, China). Methanol and ethanol were provided by Merck Serono Co., Ltd. (Beijing, China). Various multi-walled nanotubes (MWCNTs), such as carboxylic CNTs (CNTs-COOH, 98%, OD 20–30 nm, L 5–30 μm), hydroxylated CNTs (CNTs-OH, 97%, OD 30–50 nm, L 10–30 μm), amino CNTs (CNTs-NH_2_, 98%, OD 18–25 nm, L 50 μm), and CNTs (98%, OD 50 nm, L 10 μm) were supplied by XFNANO Materials Technology Company (Nanjing, China). Ultrapure water used in the study was obtained from a Milli-R04 purification system (Millipore, Germany). Paraffin oil was obtained from Sinopharm Chemical Reagent Company (Wuhan, China). 2-Methylimidazole (2-MIM, 98%) and cobaltous nitrate (Co(NO_3_)_2_·6H_2_O, 96%) were procured from Sigma (Milwaukee, WI, USA).

### 2.2 Apparatus and instruments

The surface morphology of the CNTs@ZIF-67 composite was investigated using a field emission scanning electron microscope (Sirion 200, Holland). TEM was performed with a transmission electron microscope (FEI TECNAI-G 20, USA). The FT-IR spectrum of the composite was obtained using a vertex spectrometer (Bruker VERTEX 70, Germany). The surface area and pore volume of the composite were detected in the Brunauer–Emmett–Teller (BET) mode by RMIT Applied Chemistry (ASAP 2010, Micromeritics, USA). Electrochemical information was recorded with CHI 660 C electrochemical workstations obtained from Shanghai CH Instruments. A three-electrode system was utilized with the CNTs, ZIF-67, CNTs@ZIF-67, or CNTs@ZIF-67/PFDA-modified AB (CNTs/AB, ZIF-67/AB, CNTs@ZIF-67/AB, and CNTs@ZIF-67/PFDA/AB) electrode as a working electrode, with a platinum wire and saturated calomel electrode (SCE) as the counter and reference electrodes, respectively. An Agilent 1200 series HPLC-UV was used in the identification of TBBPA in environmental samples. A Waters Sunfire C_18_ (150 × 4.6 mm, 5 μm) column was employed to equip the HPLC-UV instrument. The injection volume was 20 μL, the flow rate was 1.0 mL min^−1^, and the mobile phase was methanol : water (85 : 15, v/v). The UV spectrum was recorded at 292 nm at room temperature.

### 2.3 Synthesis of CNTs@ZIF-67composite

**Scheme 1:**
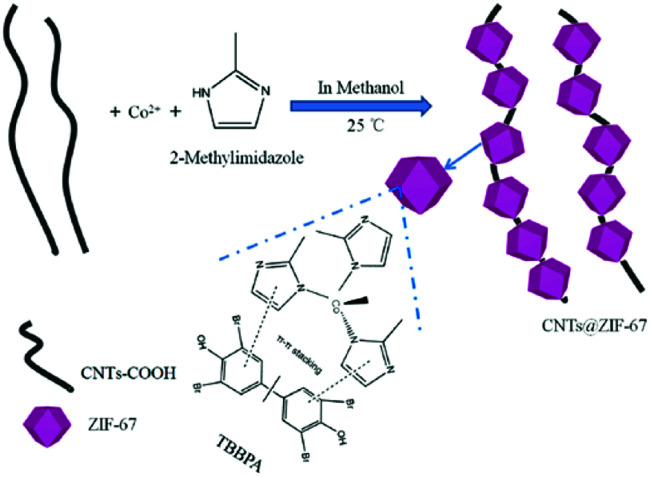
Preparation of the CNTs@ZIF-67 composite *via* a single step at room temperature.

ZIF-67 was prepared according to a previous study,^36^ and CNTs@ZIF-67 was synthesized with some modifications as shown in Scheme 1. And the details of the preparation process were written in the ESI.†

### 2.4 Fabrication of the modified electrode

The proposed electrodes were prepared by completely mixing paraffin oil (20 μL), AB (96 mg), and CNTs, ZIF-67, or CNTs@ZIF-67 (4 mg) for at least 25 min in a mortar. The mixture was firmly packed into a CP electrode holder. To ensure a reproducible working surface, butter paper was used to polish the surface of the electrode several times. The AB electrode was prepared in an identical manner, except for the omission of CNTs, ZIF-67, or CNTs@ZIF-67. PFDA (0.4 mg mL^−1^, 20 μL) was dropped onto the modified electrode, maintained at room temperature for 7 min, and washed with water. Electrode modification was accomplished after the evaporation of water from the electrode surface.

### 2.5 Electrochemical measurement

Differential pulse voltammetry (DPV) and cyclic voltammetry (CV) methods were applied to survey the electrochemical properties of the different modified electrodes. The fabricated sensor was immersed in 10 mL of a 0.1 mol L^−1^ phosphate buffer solution of pH 5.0 with different TBPPA concentrations for 10 min and then transferred to an electrochemical cell containing 10 mL of phosphate buffer solution (0.1 mol L^−1^, pH = 6). DPV curves were acquired at 0.2–0.9 V with pulse amplitude, pulse width and scan rate of 50 mV, 40 ms, and 20 mV s^−1^, respectively. The CV measurement was recorded at 0.2–0.9 V with a scan rate of 100 mV s^−1^.

Different modified electrodes were subjected to electrochemical impedance spectroscopy (EIS) recorded in potassium chloride (KCl, 0.1 mol L^−1^) containing K_3_[Fe(CN)_6_]/K_4_[Fe(CN)_6_] solution (5 mmol L^−1^). The EIS was recorded in a potential range of 0.2–0.9 V with an amplitude of 10 mV and a frequency range of 100 mHz to 10 kHz.

### 2.6 Sample pretreatment

The rain samples were obtained when the day is raining, and pool water samples were obtained from the yard of our school. The obtained four water samples were only filtered *via* hydrophobic membranes before be detected. And no other extraction processes were displayed in the pretreatment of the water samples.

## 3. Results and discussion

### 3.1 Preparation and characterization of CNTs@ZIF-67composite

Several CNTs with different functional groups, such as CNTs, CNTs-COOH, CNTs-NH_2_, and CNTs-OH, were used to prepare the CNTs@ZIF-67 composite. The adsorption capacities of ZIF-67, kinds of CNTs, and corresponding CNTs@ZIF-67 composites were determined. In general, the adsorption capacity *Q* (mg g^−1^), was calculated using the TBBPA concentration before and after adsorption by a precise amount of the different adsorbents in a constant volume of methanol. *Q* was calculated in accordance with the following equation:

1 *Q* = (*C*_0_ − *C*) *V*/*M*

Herein, *C*_0_ (mg mL^−1^) means the original TBBPA concentration; *C* (mg mL^−1^) refers to the concentration of TBBPA after adsorption, *V* (mL) means the volume of methanol, and *M* (g) means the amount of adsorbent. In Fig. S2 (see ESI†), CNTs-COOH showed the highest TBBPA adsorption capacity among the different kinds of CNTs. The adsorption capacities of the composite based on CNTs-COOH, ZIF-67, and CNTs-COOH@ZIF-67 for TBBPA were 42.55, 18.13, and 92.12 mg g^−1^, which indicated that synergistic effects were observed between CNTs-COOH and ZIF-67. Therefore, the optimum carbon nanotube for the preparation of the CNTs@ZIF-67 composite was CNTs-COOH. In our research, the stability of the adsorption capacity of the material was investigated. The adsorption experiment was processed once a week for three weeks, the obtained results of the adsorption capacity for TBBPA were in good agreement with the RSD of 5.6%. Hence, the stability of the material was satisfactory.

**Fig. 1:**
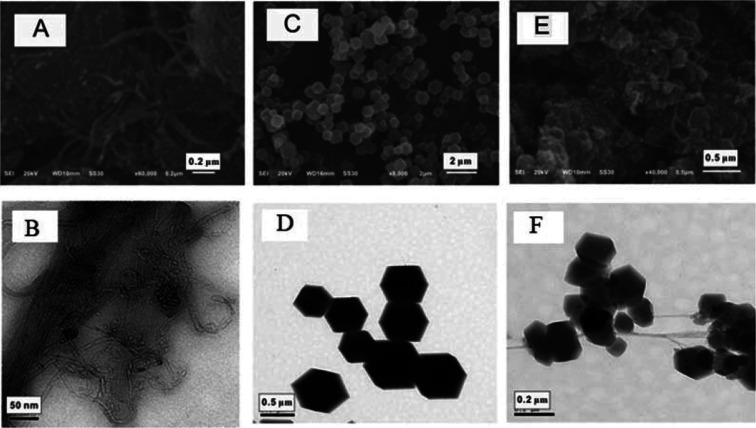
SEM and TEM images of CNTs-COOH (A and B), ZIF-67 (C and D) and CNTs@ZIF-67 (E and F).

Fig. 1 shows the morphological characteristics of CNTs-COOH, ZIF-67 and CNTs@ZIF-67. Fig. 1A and B illustrated the tubular forms of CNTs-COOH, and Fig. 1C and D presents the clear chamfered and cubic crystals of ZIF-67. In Fig. 1E and F, CNTs-COOH was wrapped with ZIF-67 in CNTs@ZIF-67 and the overall shape of the composite was similar to a bunch of grapes. The size of ZIF-67 ranged from 0.2 μm to 0.5 μm, whereas the size of ZIF-67 in the CNTs@ZIF-67 composite ranged from 0.1 μm to 0.2 μm. These phenomena suggested that the introduction of CNTs-COOH decreased the size of ZIF-67. Fig. S3 (see ESI†) shows the chemical composition of CNTs@ZIF-67 *via* XPS. It was shown that CNTs-COOH consisted of the elements C and O, while ZIF-67 and CNTs@ZIF-67 contained C, O, Co, and N. And the peaks of C, O, Co, and N in CNTs@ZIF-67 are much smaller than those in ZIF-67, these findings implied that the elemental composition was altered by the addition of CNTs-COOH, which demonstrated the successful preparation of the CNTs@ZIF-67 composite.

**Fig. 2:**
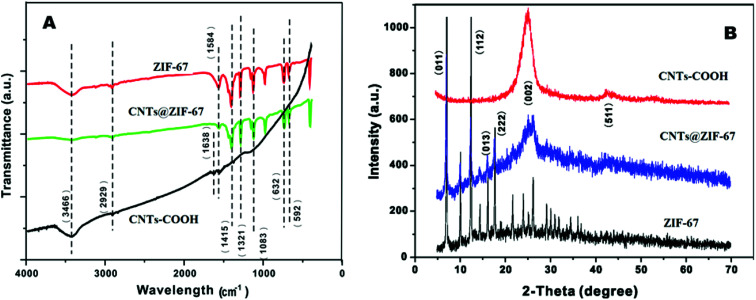
(A) FT-IR and (B) XRD spectra of CNTs-COOH, ZIF-67 and CNTs@ZIF-67.

Fig. 2 A shows the FT-IR spectra of CNTs-COOH, ZIF-67 and CNTs@ZIF-67. The appearing of the absorption peak at 1638 cm^−1^ in the CNTs-COOH spectrum confirmed the presence of carboxyl groups.^37^ FT-IR peaks in ZIF-67 and CNTs@ZIF-67 mainly arose from the 2-MIM ligand. The absorption peaks at 600–1500 cm^−1^ were derived from the bending and stretching of the imidazole ring. Additionally, the stretching modes of C=N and C–H in 2-MIM were represented by peaks at 1584 and 2929 cm^−1^. The disappearance of the carboxyl and the appearance of the peaks of ZIF-67 in CNTs@ZIF-67 proved that the fabricated CNTs@ZIF-67 composite contained the adsorption peaks of 2-MIM and CNTs-COOH, which illustrated the successful preparation of the CNTs@ZIF-67 composite.

The crystalline structures of the fabricated ZIF-67 and CNTs@ZIF-67 were confirmed by XRD spectra (Fig. 2B). The XRD pattern of the composite matched well that of ZIF-67 (ref. 36) except for the appearance of a high-intensity peak at approximately 26–27°, which corresponded to the (002) peak of CNTs-COOH.^38^ Hence, the CNTs@ZIF-67 composite was successfully prepared, and the incorporation of CNTs-COOH did not destroy the crystal structure of ZIF-67.

The surface characteristics of CNTs-COOH, ZIF-67, and CNTs@ZIF-67 were quantified using the BET test. Table 1 summarizes the Langmuir surface area (*S*_Langmuir_), surface area of BET (*S*_BET_), total micropore volume (*V*_total_), and average pore diameter (*D*_ave_) of the three materials. In Table 1, it is obvious that the total pore volume and the surface areas of CNTs@ZIF-67 composite were the largest in the three adsorbents, and the possible reason might be the addition of large amount of CNT-COOH during the preparation of CNTs@ZIF-67, which restricted the growth of ZIF-67 and resulted in the size reduction of it.

**Table 1:** BET specific surface area (*S*_BET_), Langmuir surface area (*S*_Langmuir_), total pore volume (*V*_total_), and average pore diameter (*D*_ave_) of different materials

Adsorbents	*S*_BET_ (m^2^ g^−1^)	*S*_Langmuir_ (m^2^ g^−1^)	*V*_total_ (cm^3^ g^−1^)	*D*_ave_ (nm)
CNTs-COOH	431.81	687.42	0.58	51.50
ZIF-67	507.16	592.83	0.49	47.48
CNTs@ZIF-67	1473.37	1930.56	0.71	56.25

### 3.2 Electrochemical behavior of TBBPA

**Fig. 3:**
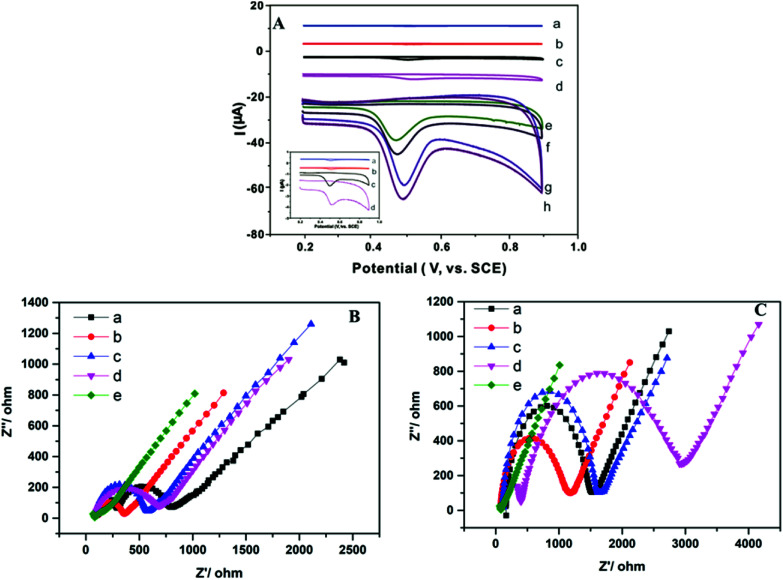
(A) CV curves of TBBPA (2.0 μmol L^−1^) on AB (a), CNTs/AB (b), ZIF-67/AB (c), CNTs@ZIF-67/AB (d), PFDA/AB (e), CNTs/PFDA/AB (f), ZIF-67/PFDA/AB (g) and CNTs@ZIF-67/PFDA/AB (h) electrodes in phosphate buffer solution (0.1 mol L^−1^, pH = 6.0). (B) EIS curves of AB (a), CNTs/AB (b), ZIF-67/AB (c), CNTs@ZIF-67/AB (d) and CNTs@ZIF-67/PFDA/AB (e) electrodes in 5 mmol L^−1^ K_3_[Fe(CN)_6_]/K_4_[Fe(CN)_6_] dissolved in KCl (0.10 mol L^−1^) before TBBPA (0.5 μmol L^−1^) extraction on. (C) EIS curves of AB (a), CNTs/AB (b), ZIF-67/AB (c), CNTs@ZIF-67/AB (d) and CNTs@ZIF-67/PFDA/AB (e) electrodes in 5 mmol L^−1^ K_3_[Fe(CN)_6_]/K_4_[Fe(CN)_6_] dissolved in KCl (0.10 mol L^−1^) after TBBPA (0.5 μmol L^−1^) extraction on. Conditions: potential scan range of 0.2–0.9 V and scan rate of 100 mV s^−1^.

Fig. 3A showed the cyclic voltammograms of different electrodes, which was recorded in 0.1 mol L^−1^ phosphate buffer solution (pH 6.0). The electrochemical response of TBBPA on the electrode was irreversible, and the only oxidized peak was formed at 0.50 V. Irreversible oxidization arose from the direct oxidization of the phenolic hydroxyl group in TBBPA.^39^ After the AB electrode was modified with ZIF-67, CNTs-COOH, and CNTs@ZIF-67, the peak current of TBBPA on the electrode was gradually increased. The largest current response was observed in the CNTs@ZIF-67 modified electrode, indicated that CNTs@ZIF-67 possessed the best adsorption ability toward TBBPA. After the electrodes were further modified with PFDA, a kind of surfactant, the current response of these electrodes to TBBPA increased further. This because the PFDA modified on the electrode can adsorbed more TBBPA onto the electrode *via* hydrophoic reaction and hydrogen-bond. Surfactants, which contain a hydrophilic head and a hydrophobic end, have been used to develop electrochemical sensors for enhancing the sensitivity of electrodes.^40^ However, PFDA has yet to be utilized for electrochemical sensors. The potential mechanism at the oxidation peak in cyclic voltammetry was studied and the electrochemical reaction process of TBBPA was investigated at CNTs@ZIF-67/PFDA/AB electrode. Fig. S4A† showed the oxidation peak current of TBBPA was linear to the scan rate in the range of 20 mV s^−1^ to 120 mV s^−1^ and the equation was *i*_p_ = 0.035*ν* + 0.214 (*R*^2^ = 0.9808), which revealed that the oxidation of TBBPA at CNTs@ZIF-67/PFDA/AB electrode was a surface-controlled process. Meanwhile, *E*_p_ has a linear relation with Napierian logarithm of *ν*(ln *ν*) in the range of 20 mV s−1 to 120 mV s^−1^ (Fig. S4B†) with the equation of *E*_p_ = 0.076*ν* + 0.356 (*R*^2^ = 0.9857). Regarding the totally irreversible electrode process and a surface-controlled^41^*E*_p_ could be defined by the following eqn (2):

2 *E*_p_ = *E*^0^ + *RT*/(*αnF*) ln[*RTk*^0^/(*αnF*)] + *RT*/(*αnF*) ln ν

In which *E*^0^ is the formal potential, *T* is the temperature, *α* is the transfer coefficient, *n* is the number of the electron transfer, *F* is the Faraday constant, *k*^0^ is the electrochemical rate constant and *ν* is scan rate and *αn* is calculated to be 1.26. Generally, in irreversible electrode process, *α* is set to be 0.5. Thus, the electron transfer number (*n*) was nearly 2 in this research. The electro-oxidation of TBBPA at SB3-16/ABPE was suggested to be a two-electron and two-proton process since the number of electron was equal to that of proton.

The EIS method was employed to characterize the resistance of the modified electrode with K_3_[Fe(CN)_6_]/K_4_[Fe(CN)_6_] as indicator. The impedance spectra contained both a linear part and a semicircular part. At low frequencies, the linear part is assigned to diffusion, and at high frequencies, the semicircular part is related to the limited transfer of electrons. Therefore, the electron transfer resistance can be indicated by the diameter of the semicircle. Fig. 3B shows the EIS results of the proposed electrodes. Before the adsorption of TBBPA, the diameter of the semicircle of different electrodes exhibited the following pattern: AB electrode > ZIF-67/AB electrode > CNTs@ZIF-67/AB electrode > CNTs/AB electrode > CNTs@ZIF-67/PFDA/AB electrode. This finding suggested that the incorporation of CNTs-COOH in CNTs@ZIF-67 improved the conductivity of ZIF-67, which was in agreement with the result of the reported research.^42^ In Fig. 3C, after the adsorption of TBBPA, the semicircle diameter of these modified electrodes decreased in the following order: CNTs@ZIF-67/AB electrode, ZIF-67/AB electrode, CNTs/AB electrode, AB electrode and CNTs@ZIF-67/PFDA/AB electrode. Therefore, CNTs@ZIF-67 modified electrode displayed the largest semicircle diameter, this was because it has the largest adsorption capacity toward TBBPA among these adsorbents. What was more, the CNTs@ZIF-67/PFDA/AB modified electrode yielded the smallest semicircle diameter both before and after TBBPA was adsorbed. This might because the PFDA modified on the electrode could adsorb more K_3_[Fe(CN)_6_]/K_4_[Fe(CN)_6_] onto the surface of the sensor, which increased the electron transfer of the electrode.

### 3.3 Parameters affecting TBBPA detection

#### 3.3.1 Effects of the electrode composition

**Fig. 4:**
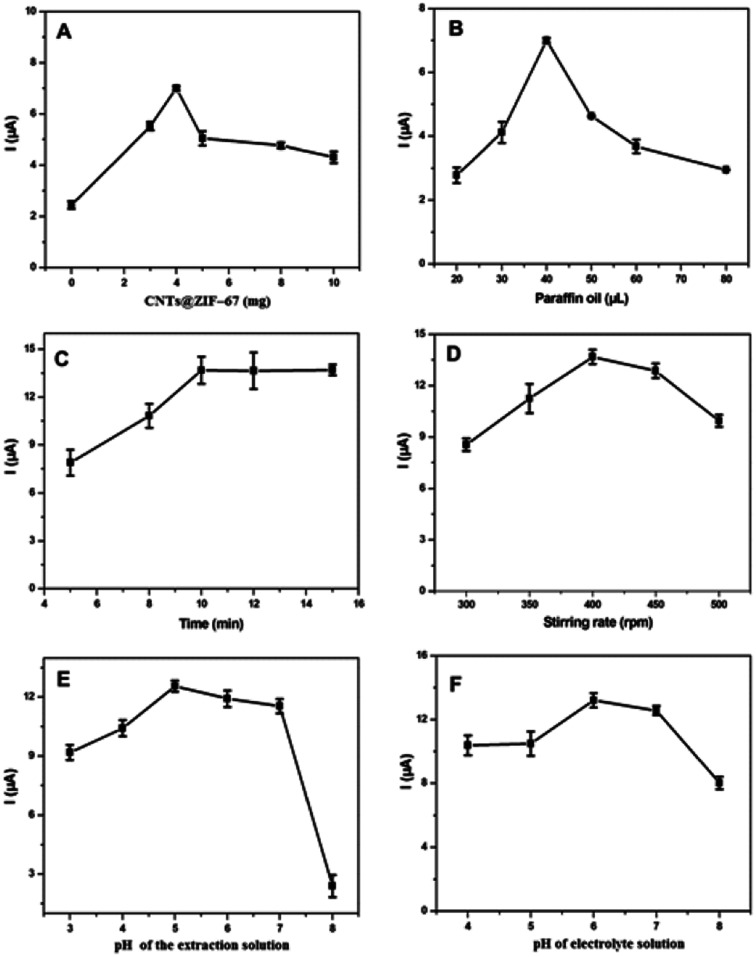
Parameters affecting the peak current of TBBPA on the CNTs@ZIF-67/PFDA/AB electrode, in which the following parameters were optimized: (A) amounts of CNTs@ZIF-67; (B) the volume of paraffin oil; (C) the extraction time; (D) the stirring rate; (E) the pH value of the extraction solution; and (F) the pH value of the electrolyte solution. DPV method conditions: scan rate of 20 mV s^−1^, pulse width of 40 ms, and pulse amplitude of 50 mV.

Different amounts of CNTs@ZIF-67 in the AB paste were applied to investigate the effect of the amount of CNTs@ZIF-67 on the current response to TBBPA. In Fig. 4A, the highest TBBPA response was obtained when the amount of CNTs@ZIF-67 was 4 mg. The TBBPA adsorption efficiency on the electrode surface increased when the amount of CNTs@ZIF-67 increased from 0 mg to 4 mg. Conversely, the catalytic ability of the electrode and the current response to TBBPA decreased when the amount of CNTs@ZIF-67 was more than 4 mg. The effect of the volume of paraffin oil on the peak current of TBBPA was also studied (Fig. 4B). When the volume of paraffin oil was less than 20 μL, the surface of the electrode was neither repeatable nor durable because of its poor physical property. When the volume of paraffin oil was greater than 20 μL, the current response to TBBPA also decreased because the increasing amount of the insulating paraffin oil decreased the electrode conductivity. Thus, the optimum condition for paraffin oil was 20 μL in this study.

Fluorinated surfactants (FSs), a subset of fluorochemicals, have been widely used in commercial production.^43^ However, FS-based electrochemical sensors have yet to be developed. Fig. 3 illustrates the potential of FSs in the construction of electrochemical sensors. In this study, various fluorinated surfactants, including PFUA, PFOA, PFNA, PFDA, PFUnDA, PFDoDA and PFTA were used to investigate the influence of the C–F chain length on the peak current of TBBPA. When the C–F chain length of the FS was 10, the current response can obtain the best effect. The reason and figure are shown in Fig. S5 (see ESI†). Therefore, PFDA was chosen for our subsequent analysis. The influence of the different hydrophilic ends of the FSs on the current response to TBBPA was also investigated (Fig. S6A (see ESI†)). It was clear that the peak currents of TBBPA for the electrodes modified with PFD-OH and PFD-SO_3_ were higher than that for the modified electrode without FSs. This may be because the interaction between TBBPA and the hydroxyl or sulfonic acid ends of the fluorinated surfactants increased the amount of TBBPA on the electrode surface. However, the current response increased remarkably when PFD-COOH (PFDA) was introduced during the modification of the electrode. This result demonstrated that the amount of TBBPA adsorbed onto the electrode was greatest when the FS ended with a carboxyl group. In this study, the preconcentration of TBBPA on the electrode was affected by the hydrophobic phase and the hydrophilic ends of the FSs, and the optimal FS in this study was PFDA.

Both the concentration and volume of PFDA were investigated. The influence of the concentration of PFDA on the current response to TBBPA is shown in Fig. S6B (see ESI†). The current response to TBBPA initially increased as the concentration of PFDA increased, but decreased when the concentration of PFDA was greater than 0.4 mg mL^−1^. The influence of the volume of PFDA on the current response was examined in Fig. S6C (see ESI†). As the volume of PFDA increased, the current response to TBBPA was remarkably enhanced. The current response to TBBPA increased gradually when the volume of PFDA was lower than 20 μL. Therefore, the optimum concentration and volume of PFDA were 0.4 mg mL^−1^ and 20 μL, respectively.

#### 3.3.2 Effect of extraction conditions

The parameters affecting the extraction efficiency of TBBPA were investigated, including the stirring rate, extraction time, and pH of the extraction solution. Fig. 4C illustrates the effect of extraction time on the peak current of TBBPA. As the extraction time was prolonged, the current response of TBBPA remarkably increased. However, when the extraction time was longer than 10 min, the peak current of TBBPA only increased slowly. Thus, the optimum extraction time for TBBPA detection was 10 min. The influence of the stirring rate on the peak current of TBBPA was examined in the range of 300–500 rpm (Fig. 4D). When the stirring rate was increased to 400 rpm, the current response improved gradually. However, when the stirring rate was higher than 400 rpm, the current response of TBBPA remained constant. Therefore, in the subsequent analyses, a stirring rate of 400 rpm was chosen. In Fig. 4E, the current response to TBBPA of the prepared CNTs@ZIF-67/PFDA/AB electrode increased gradually as the pH of the adsorption solution increased from 3 to 5. However, the electrochemical current of TBPPA decreased when the value of pH was further increased, which indicated that the activity of TBBPA on CNTs@ZIF-67/PFDA/AB electrode was pH dependent. Therefore, pH 5 was selected for the extraction solution used to detect TBBPA.

#### 3.3.3 Effect of electrochemical measurement parameters

DPV was selected to develop a highly sensitive sensor. The investigation primarily included certain electrochemical parameters, including the pH value of the electrolyte solution and the parameters of DPV method. The influence of the pH value of the electrolyte solution on the current response to TBBPA was investigated in a pH range of 3–8 (Fig. 4F). The largest current response to TBBPA was obtained when the pH value was 6. It was apparent that the optimal pH value of the electrolyte solution was below the p*K*_a_ of TBBPA (p*K*_a1_ = 7.5, p*K*_a2_ = 8.5 (ref. 44)), which indicated that the un-dissociated TBBPA can be adsorbed onto the sensor much more easily than its dissociated form.^45^ Therefore, to increase the current response to TBBPA, the optimum pH value of the electrolyte solution was 6.

The main DPV parameters, including the pulse amplitude, scan rate and pulse width, were investigated in the ranges of 20–200 mV, 10–30 mV s^−1^ and 20–100 ms, respectively. The results indicated that the best peak pattern was formed when the pulse amplitude, scan rate and pulse width were 50 mV, 20 mV s^−1^ and 40 ms, respectively.

### 3.4 Interferences, reproducibility and stability

The influence of the organic substances and inorganic ions in environmental water samples was tested. When different concentrations of the interferents were absent or present, the current responses of 0.5 μmol L^−1^ TBBPA were both recorded by the proposed sensor. The tolerance level was defined as the ratio of interferent concentration to TBBPA concentration (0.5 μmol L^−1^) when the interferent caused a deviation of ±5% in the current intensity for TBBPA. The obtained results demonstrated that 200-fold concentrations for Al^3+^ and Ca^2+^, 300-fold for K^+^, Mg^2+^, and Ca^2+^, 500-fold for SO_4_^2−^ and CO_3_^2−^, 600-fold for NO_3_^−^ and Cl^−^, and 1000-fold for Na^+^ did not influence the determination of TBBPA. For organic substances, 10-fold concentrations of TBBME and TBBDE, 5-fold for BPAF and BPA, 2-fold for TCBPA and TBBPS as TBBPA analogs did not affect the detection of TBBPA. Therefore, the performance of the developed sensor in the presence of these interferents was acceptable.

The reproducibility and stability of the developed sensor were investigated under optimum conditions. Excellent reproducibility was observed in nine parallel electrodes with a relative standard deviation (RSD) of 3.59%. Good inter-day stability with an RSD of 5.32% was observed after the CNTs@ZIF-67/PFDA/AB electrode was subjected to four assays each day for 2 weeks. Satisfactory intra-day stability with an RSD of 4.82% was detected by the same electrode after nine experiments within 1 day.

### 3.5 Analytical characterization

**Fig. 5:**
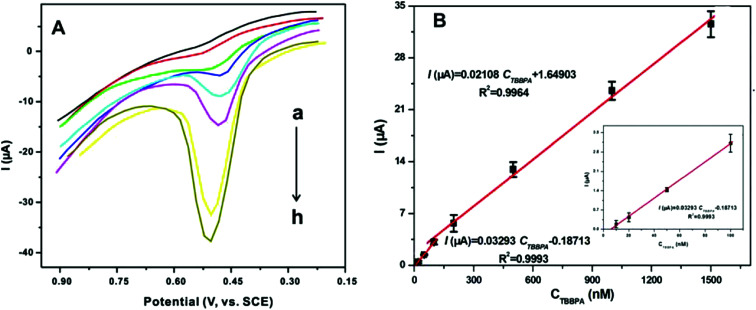
(A) DPV patterns of TBBPA on CNTs@ZIF-67/PFDA/AB electrode at the concentrations of (a–h): 0.01, 0.02, 0.05, 0.1, 0.2, 0.5, 1.0 and 1.5 μmol L^−1^ and (B) linear curves between TBBPA concentrations and the current responses by the developed sensor under the optimum conditions.

To investigate the analytical performance of the CNTs@ZIF-67/PFDA/AB electrode, the calibration curves for the detection of TBBPA were drawn using DPV in a phosphate buffer solution (0.1 mol L^−1^) under the optimal conditions. In Fig. 5A, the current response increased with the TBBPA concentration ranging from 0.01 to 1.5 μmol L^−1^. Two linear relationships were shown between the current responses and TBBPA concentrations (Fig. 5B). The linear equation was *I* (μA) = 0.03293*C*_TBBPA_ (μmol L^−1^) − 0.1871 (*r*^2^ = 0.9993) at concentrations between 0.01 and 0.10 μmol L^−1^ and *I* (μA) = 0.02108*C*_TBBPA_ (μmol L^−1^) + 1.6490 (*r*^2^ = 0.9964) at concentrations between 0.1 and 1.5 μmol L^−1^, where *C*_TBBPA_ refers to the TBBPA concentration. The limit of detection (LOD) of the sensor was calculated using the relation *ks*/*b*, where *k* is a constant parameter (*k* = 3), *s* refers to the standard deviation obtained from the peak current for TBBPA (0.01 μmol L^−1^, *n* = 12), and *b* denotes the slope of the first calibration curve in the TBBPA determination. Ultimately, the LOD of the developed sensor was calculated to be 4.23 nmol L^−1^. To evaluate the sensitivity of the prepared sensor, the comparison of this sensor with other reported sensors for TBBPA determination is shown in Table 2. From Table 2, it is apparent that the sensitivity of the prepared sensor in this work was quite satisfactory.

**Table 2:** Comparison of analytical characterization of reported sensors for the detection of TBBPA

Sensors	Linear range (μmol L^−1^)	Detection limit (nmol L^−1^)	Reference
DODMA^a^/GCE	0.0018–0.92	1.05	46
CTAB/NG-TPA^b^/GCE	0.01–1	9.00	17
MMIP^c^/CPE	0.005–2	0.77	47
GNRs/poly-Cys^d^/GCE	0.01–10	3.20	48
AB/GCE	0.018–634	11.00	15
CNTs@ZIF-67/ABE	0.01–1.5	4.23	This work

a Dioctadecyldimethylammonium bromide.

b Hexadecyltrimethylammonium bromide/nitrogen-doped graphene-1,3,6,8-pyrenetetrasulfonic acid tetrasodium salt.

c Magnetic molecularly imprinted polymer.

d Au nanorods/poly-cysteine.

### 3.6 Application to environmental water samples

To predict the practical applications of the proposed sensor, environmental water samples spiked with different concentrations of TBBPA standards were analyzed using the fabricated sensor. In Table 3, each sample was evaluated in triplicate. The RSD of the current responses was less than 6.02%, which indicated the good reproducibility of the sensor. To verify the accuracy of the sensor, HPLC-UV was employed to detect TBBPA in the same samples. It was clear that the results of the HPLC-UV method were consistent with those of the proposed electrochemical method. Therefore, the accuracy of the developed CNTs@ZIF-67/PFDA/AB sensor was excellent and its application to environmental water samples was reliable and effective.

**Table 3:** Detection of TBBPA in environmental water samples by the proposed electrochemical sensor (*n* = 3) and HPLC-UV method (*n* = 3)

Sample	Added (μmol L^−1^)	Electrochemical			HPLC-UV		
		Found (μmol L^−1^)	Recovery (%)	RSD (%)	Found (μmol L^−1^)	Recovery (%)	RSD (%)
Rain 1	0.02	0.0189	94.97	0.81	0.0217	108.78	1.30
	0.1	0.1066	106.62	3.07	0.1112	111.16	1.01
	0.5	0.5056	101.12	1.78	0.4924	98.40	2.06
Rain 2	0.02	0.0191	95.52	2.56	0.0207	103.72	3.76
	0.1	0.1040	104.04	2.41	0.1026	102.64	2.89
	0.5	0.5309	106.18	4.13	0.4931	98.62	4.32
Pool water 1	0.02	0.0186	93.22	1.45	0.0194	796.9	2.08
	0.1	0.0929	92.89	2.40	0.1054	105.43	0.76
	0.5	0.5339	106.78	1.10	0.4894	97.87	2.36
Pool water 2	0.02	0.0208	104.19	0.81	0.0192	96.13	3.68
	0.1	0.1067	106.68	2.17	0.1093	109.31	3.21
	0.5	0.5259	105.20	6.02	0.5089	101.79	1.20

## 4. Conclusion

The conductive CNTs@ZIF-67 composite was easily prepared. The adsorption capacity of the prepared composite for TBBPA was as great as 92.12 mg g^−1^. A novel AB paste electrode modified with this composite and PFDA was developed to detect TBBPA in environmental water samples. The proposed sensor showed excellent stability and reproducibility in the detection of TBBPA with an LOD of 4.23 nmol L^−1^. Finally, the developed sensor was employed to determine TBBPA in environmental water samples, and the results were in good agreement with those recorded using HPLC-UV. Thus, the present work demonstrated the promising application of CNTs@ZIF-67 modified sensors in environmental analysis.

## Conflicts of interest

The authors declare that they have no conflicts of interests.

## Supplementary Material

RA-010-C9RA06980A-s001
