# Supplementary material for: Electrochemical determination of tetrabromobisphenol A in water samples based on a carbon nanotubes@zeolitic imidazole framework-67 modified electrode
Source: RSC Adv. 2020 Jan 10;10(4):2123–32. doi: 10.1039/c9ra06980a (PMC9048974; doi:10.1039/c9ra06980a)
Supplement: RA-010-C9RA06980A-s001 [file RA-010-C9RA06980A-s001.pdf]

Electronic Supplementary Material (ESI) for RSC Advances.
This journal is © The Royal Society of Chemistry 2019

Electrochemical determination of tetrabromobisphenol A in water samples based on carbon nanotubes@zeolitic imidazole framework-67 modified electrode

Tingting Zhou[a,1], Xiaoya Zhao[b,1], Yinghua Xu[a] , Yun Tao[c], Dan Luo[c], Liqin Hu[c], Tao Jing[c], Yikai Zhou[c], Peng Wang[b]*, Surong Mei[c]

[a] *Department of Clinical Laboratory, The Affiliated Hospital of Qingdao University, #16, Jiangsu Road, Qingdao, Shandong, 266003, China*

[b] *Technology Center of Wuhan Customs District, # 588 Qingtaidadao Road, 430022, Wuhan, China*

[c] *State Key Laboratory of Environment Health (Incubation), Key Laboratory of Environment and Health, Ministry of Education, Key Laboratory of Environment and Health (Wuhan), Ministry of Environmental Protection, School of Public Health, Tongji Medical College, Huazhong University of Science and Technology, #13 Hangkong Road, Wuhan, Hubei, 430030, China*

*** Corresponding authors:**

Peng Wang

E-mail: 18681533@qq.com

Address: Technology Center of Wuhan Customs District, No.588 Qingtaidadao Road, 430022, Wuhan, China

[1]These authors equally contributed to this work

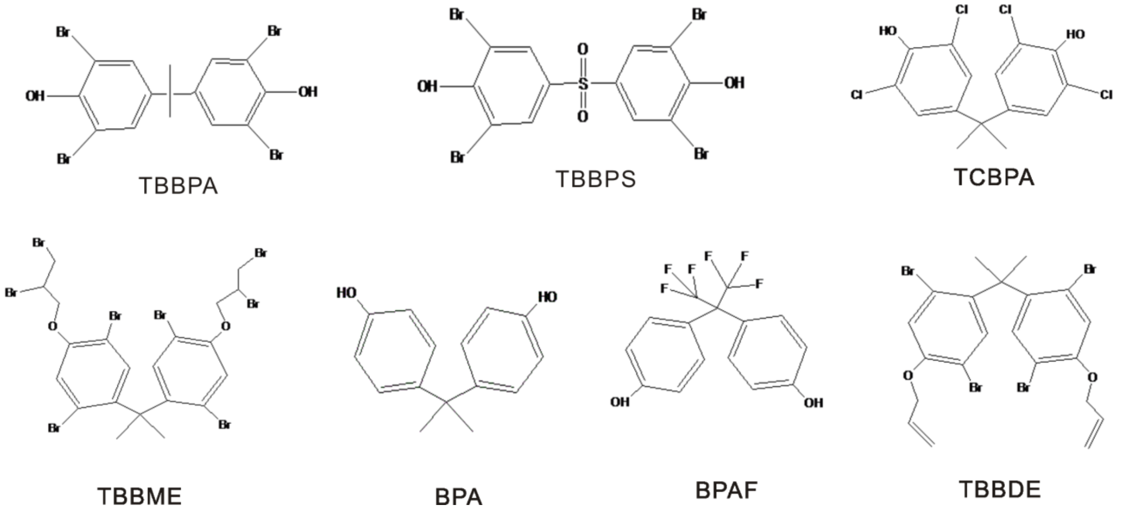

Fig. S1. Chemical structures of TBBPA and Its analogues.

Experimental section

The preparation process of the new material:

CNTs-COOH (200 mg) was dispersed in methanol (100 mL) by ultrasonication for 10 min, and 8 mmol of 2-MIM was added to the well-dispersed solution of CNTs-COOH. In all, 4 mmol of $Co(NO_3)_2{\cdot}6H_2O$ was placed in another container and dissolved in methanol (100 mL). The resulting pink solution of $Co(NO_3)_2{\cdot}6H_2O$ was slowly added dropwise to the 2-MIM and CNTs-COOH solution with mechanical stirring at 330 rpm and room temperature. The dropwise addition was stopped after 20-30 min, and the reaction was completed after stirring for 2 h. The precipitates thus obtained were collected through centrifugation, and then washed several times with ethanol. Finally, the obtained precipitates were dried at 60 °C under vacuum for 6 h, then at 100 °C for another 12 h. Other ZIF-composites based on CNTs, $CNTs\text{-}NH_2$, CNTs-OH were prepared in the same manner.

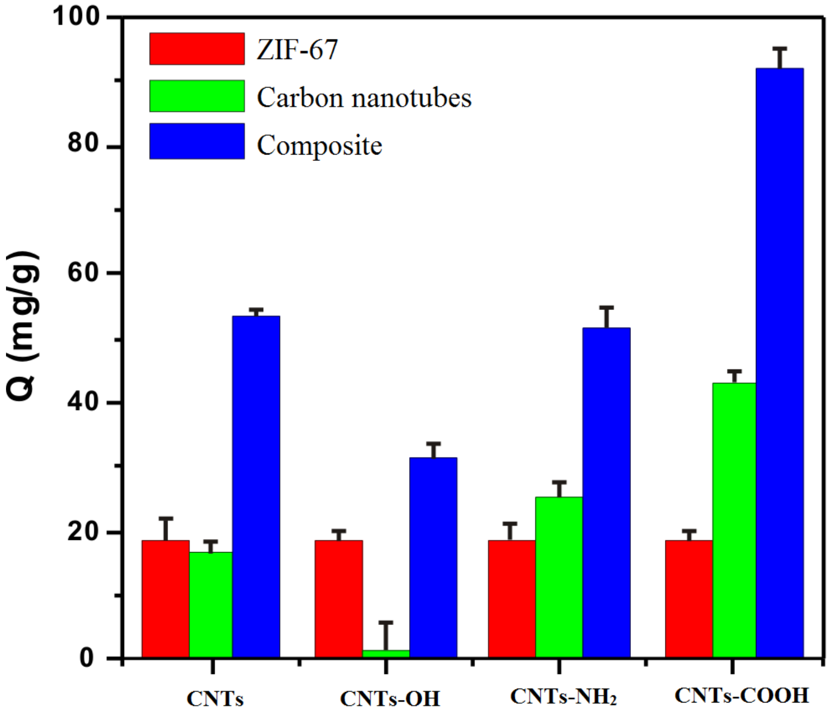

Fig. S2. The adsorption capacity of different kinds of adsorbents for TBBPA, including ZIF-67, various carbon nanotubes with different functional groups and the carbon nanotubes@ZIF-67 composites. ($M_{adsorbent}$ is 20 mg, C_0 is 2.0 mg mL^{-1}).

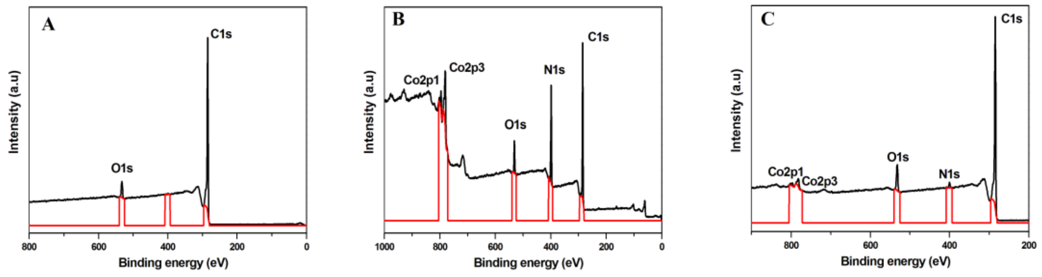

Fig. S3. XPS spectra of CNTs-COOH (A), ZIF-67 (B) and CNTs@ZIF-67(C).

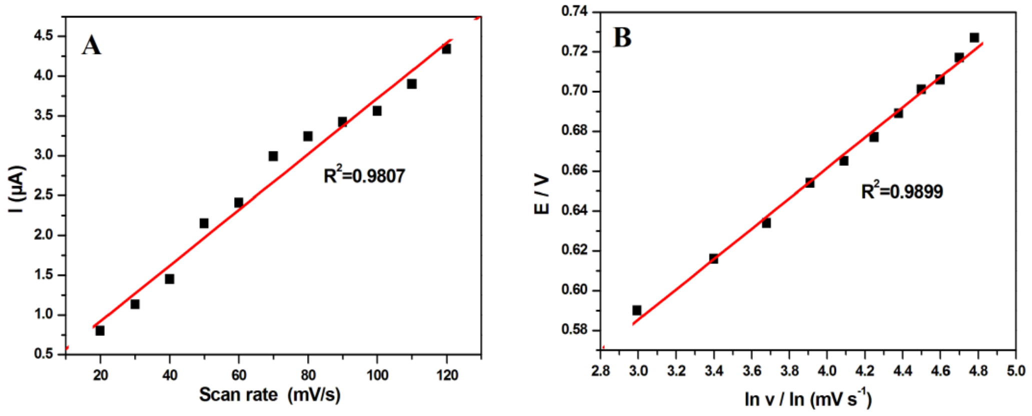

Fig. S4. (A) The linear relationship of peak current and scan rate and (B) The polt of peak potential of peak current and natural logarithm of scan rate.

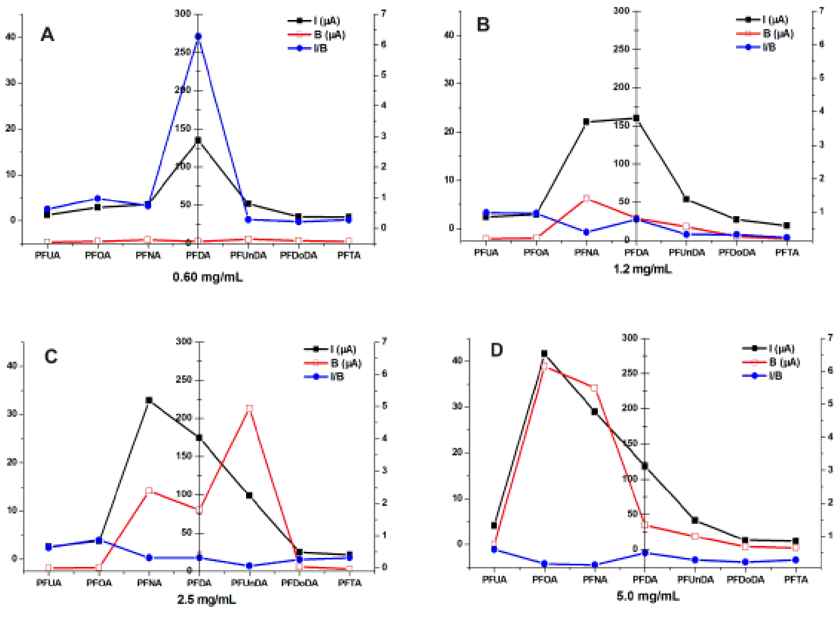

Fig. S5. The response of different fluorinated surfactant with different chain length.

In Fig. S5, I means the current response of the electrode, B means the baseline of the electrochemical reaction, and I/B refers to the ratio of the current response to the baseline of the electrochemical response. The lager the I/B is, the lower the detection limit will be. As can be seen in the figure below, when the concentration of the

fluorinated surfactant was decrease gradually, the I/B of PFDA was increased gradually, therefore, PFDA was chosen in this research.

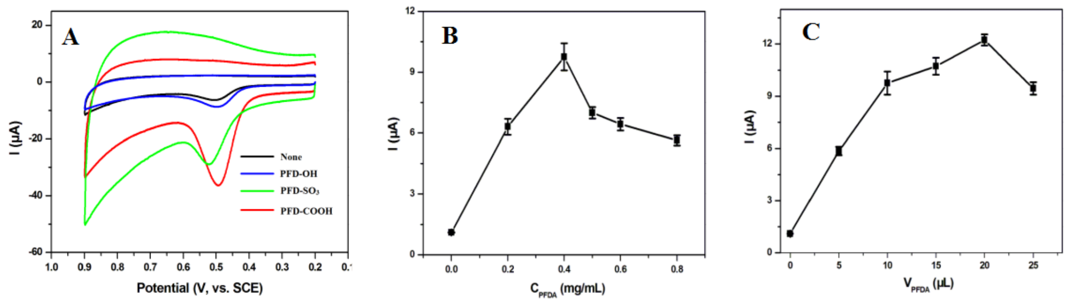

Fig. S6. (A) CV current responses of 0.5 μmol L^{-1} TBBPA at CNTs@ZIF-67/AB electrode in the presence of various fluorinated surfactants with different hydrophilic groups at a concentration of 0.6 mg mL^{-1}. Other conditions are identical to those of Fig. 3. (B) and (C) are the effects of the concentration and the volume of PFDA on the current response to TBBPA by the CNTs@ZIF-67/PFDA/AB electrode. Other conditions are in keeping with those in Fig. 4.
